# Mechanical and Lubrication Properties of Double Network Ion Gels Obtained by a One-Step Process

**DOI:** 10.3390/ma15062113

**Published:** 2022-03-13

**Authors:** Hiroyuki Arafune, Yuma Watarai, Toshio Kamijo, Saika Honma, Takaya Sato

**Affiliations:** Department of Creative Engineering, National Institute of Technology, Tsuruoka College, 104 Sawada Ino-oka, Tsuruoka 997-8511, Yamagata, Japan; s180018@edu.tsuruoka-nct.ac.jp (Y.W.); kamijo@tsuruoka-nct.ac.jp (T.K.); saika@tsuruoka-nct.ac.jp (S.H.)

**Keywords:** ionic liquid, double network gel, one-pot synthesis, tribology

## Abstract

Human joints support us to reduce the impact on our body and move them smoothly. As they are composed of gel-like structures, gel materials with soft and resilient properties are expected, as lubricants, to provide high efficiency and a long lifetime for mechanical parts. While double network gels including ionic liquids as swelling agents possess high mechanical strength and stable low friction under high temperature or vacuum, their fabrication process is complex and time-consuming. In this study, we applied one-pot synthesis to a double network ion gel (DNIG) to obtain a thin gel film by a simple coating method and examined its thermal, mechanical and tribological properties. The DNIG was obtained by one-pot synthesis (DNIG-1) combining polycondensation of tetraethoxysilane and radical polymerization of methyl methacrylate to form silica and poly(methyl methacrylate) as a 1st and 2nd network, respectively. Such obtained DNIG-1 was characterized and compared with DNIG obtained by a conventional two-step process (DNIG-2). Thermogravimetric analysis and the compressive stress–strain test showed high thermal stability and mechanical strength of DNIG-1. As friction at the glass/DNIG-1 interface showed high friction compared with that at glass/DNIG-2, various counterface materials were applied to examine their effect on the friction of DNIG-1. As SUS304/DNIG-1 showed much lower friction compared with glass/DNIG-1, the difference in the friction was presumably due to the different adsorption forces and compatibility between the materials.

## 1. Introduction

Friction is a ubiquitous phenomenon that is often seen in everyday life. After getting up in the morning, we rub the sleep from our eyes, eat breakfast and brush our teeth, where friction is included in all of these motions. In these motions, human cartilages support us to reduce the impact on human joints and move smoothly [1]. They are composed of gel-like structures with collagen fiber backbones and macromolecules, proteins and phospholipids, and can maintain low friction coefficients in the range 0.001–0.01 under tens of MPa for several tens of years without replacement [2]. Gel materials have fascinated the researchers of tribology who study the principle and application of friction, lubrication and wear to invent novel lubricants with soft and resilient properties [3,4,5]. Such development of soft and resilient tribomaterials (SRT materials [6]) is distinguished from the conventional process to obtain hard materials such as diamond-like carbon (DLC). Hard coatings can reduce friction based on the idea of reducing real contact area; furthermore, such a hard surface has the potential risk to hurt contact materials and induce severe wear and increase friction immediately [7]. Thus, SRT materials are expected, as a novel lubricant strategy, to provide high efficiency and a long lifetime for mechanical parts where low friction is maintained under high pressure without abrasive wear of counterface materials.

Double network hydrogels (DN hydrogels) based on a brittle 1st gel network and resilient 2nd gel network possess both high solvent content (mostly >80%), high mechanical strength and low friction, whose physicochemical properties can be tuned by selecting an appropriate polymer backbone with various properties [8]. Based on their high mechanical strength and toughness [9], high water content and lubricious surface [10], they are expected to be used as artificial biomaterials [11]. However, as they are easily dried, losing their surface characteristics, under harsh conditions such as long time exposure, high temperature or vacuum, their industrial application is still limited without substituting water for a hardly volatile solvent [12].

One effective approach to overcome such disadvantages of hydrogels is to substitute water with ionic liquids (ILs) to obtain ion gels [13]. ILs are molten salts wholly composed of cations and anions whose melting temperatures are lower than 100 °C [14]. Thanks to the high ionic conductivity, thermal stability and negligible volatility of ILs, ion gels have attracted much attention for various research fields such as electrochemical devices, lubricants, CO_2_ separation membrane, and so on [15,16,17]. Our research group has developed double network ion gels (DNIGs) where the polymer backbone and swelling agent are composed of ILs. We applied an IL, *N*,*N*-diethyl-*N*-(2-methoxyethyl)-*N*-methylammonium bis (trifluoromethylsulfonyl)amide (DEME-TFSA) as a swelling agent and lubricant, IL-type polymer composed of the derivative of DEME-TFSA with a polymerizable moiety, *N*,*N*-diethyl-*N*-(2-methacryloylethyl)-*N*-methylammonium bis (trifluoromethylsulfonyl)amide (DEMM-TFSA) as a 1st network and methyl methacrylate (MMA) as a 2nd network. DNIG showed not only high compressive fracture stress (30 MPa) with high solvent content (85 wt%), but also could maintain low friction under high temperature (80 °C) or vacuum (2.4 × 10^−4^ Pa) [18]. These results showed that DNIGs can be applied as robust gel lubricants under harsh conditions where DN hydrogels are difficult. For example, as a conventional rubber seal scarifies lubrication property instead of its sealing properties, DNIGs are expected to be utilized as a novel sealing gel lubricant possessing both sealing and lubrication properties under high temperature or vacuum, which is applicable in outer space.

Despite these advantages, the fabrication process of DNIG is complex and time-consuming because the conventional stepwise photoradical polymerization process was adapted. In this process, the precursor solution of the 1st network is polymerized by UV irradiation or heat to obtain the 1st gel. Second, the obtained 1st gel is immersed into a precursor solution of the 2nd network until it reaches swelling equilibrium, followed by UV irradiation or heat to obtain a DN gel. The swelling speed of the 1st gel is determined by the cooperative diffusion and its time length is proportional to the square of its thickness. Thus, tensile stress is induced by immediate swelling in the case of thin gel film, leading to breaking itself. Songmio et al. fabricated ultrathin DN hydrogels of poly (2-acrylamide-2-methylpropane sulfonic acid)/polyacrylamide (PAMPS/PAAm) of ~30 µm by controlling swelling behavior using the salt effect and the pre-reinforced technique [19,20]. They immersed the thin PAMPS gel into a precursor solution of AAm containing 0.08 M NaCl to avoid fracture of the thin PAMPS gel film by reducing osmotic pressure. Such obtained PAMPS gel was pre-reinforced with PAAm and can be fully stretched in AAm solution without salt to obtain a thin film of PAMPS/PAAm hydrogel. However, the complexity of the fabrication process remains in such a multi-step approach.

For the industrial application of DNIG, the development of a one-pot synthesis process is effective due to its simplicity and low cost. Such obtained gel film lubricant can easily be attached to mechanical parts by a simple fixation method. This process opens up the way to fabricate thin gel films by a simple coating method, which possesses both lubricity and sealing performance. However, radical polymerization of both 1st and 2nd networks at the same time results in the formation of random polymers, losing characteristics of the DNIG. Recently, Kamio et al. reported the one-pot synthesis of DNIG by combining polycondensation of tetraethoxysilane (TEOS) and radical polymerization of dimethyl acrylamide. Such independent reaction prevents the reaction between the 1st and 2nd monomer, which led to the one-pot synthesis of DNIG [21]. In this case, a brittle inorganic network of silica particles contributed to the 1st network to dissipate loaded energy and gave high fracture energy for DNIG with an inorganic/organic network of TEOS and poly (dimethyl acrylamide). Such a simple one-pot process opened up the way to form free-shapeable robust ion gels, which are expected to be utilized as novel sealing gel lubricants with both sealing and lubrication properties. However, as they studied the mechanical properties and showed the robustness of DNIGs obtained by one-pot synthesis, their tribological properties are still unclear.

In this study, one-pot synthesis of DNIG combining polycondensation of TEOS and radical polymerization of MMA was examined to obtain DNIG with silica as the 1st network and poly (methyl methacrylate) (PMMA) as the 2nd network. As DNIG in a previous study composed of poly (DEMM-TFSA) and PMMA showed high mechanical strength and low friction, we also utilized PMMA to obtain such properties in this study. The thermal, mechanical and tribological properties of DNIG obtained by one-pot synthesis were obtained and compared with conventional DNIG. In the case of tribological characterization, the effect of counterface materials was also studied to examine the performance of DNIG as a gel lubricant.

## 2. Materials and Methods

### 2.1. Fabrication of DNIGs

One-pot synthesis of DNIG was performed by combining polycondensation and thermal radical polymerization. Acetonitrile was used as received from Kanto Chemical Co. Ltd. (Tokyo, Japan) *N*-(2-methoxyethyl)-*N*-methylpyrrolidiniium trifluoromethylsulfonylamide (MEMP-TFSA) was used as received from Nisshinbo HD. 2,2′-azobis(isobutyronitrile) (AIBN), formic acid, tetraethoxysilane (TEOS) and triethylene glycol dimethacrylate (TEGDMA) were used as received from Fujifilm Wako pure chemical corporation. Methyl methacrylate was purchased from Nakalai Tesque and purified to remove the polymerization inhibitor. One-pot synthesis of double network ion gels was performed by combining polycondensation and thermal radical polymerization. TEOS (8.0 wt%) as a 1st monomer, formic acid (6.5 wt%) for catalysis of the 1st network formation, MMA (22 wt%) as a 2nd monomer, AIBN (0.37 wt%) as an initiator, TEGDMA (0.87 wt%) as a crosslinker, MEMP-TFSA (57 wt%) and acetonitrile (5.5 wt%) were mixed and charged into a Schlenk tube, followed by argon bubbling for 5 min to deoxygenate the solution. The solution was poured into a reaction cell made from a pair of glass substrates separated by 0.2-mm-thick silicone rubber by using an injection syringe. The sample solution was heated at 50 °C under an Ar atmosphere for 24 h and then heated at 50 °C under vacuum for 24 h. Such thermal process was effective to promote polycondensation of TEOS and thermal radical polymerization of MMA to form the 1st network (silica) and the 2nd network (polymethyl methacrylate, PMMA) to form DNIG by a one-step process (DNIG-1).

As a control experiment, a single network gel of TEOS or MMA was also synthesized. A precursor solution of TEOS gel was obtained by mixing TEOS (19 wt%), MEMP-TFSA (58 wt%), formic acid (16 wt%) and acetonitrile (7 wt%). A precursor solution of MMA gel was obtained by mixing MMA (43 wt%), MEMP-TFSA (49 wt%), AIBN (0.73 wt%), TEGDMA (1.6 wt%) and acetonitrile (5.5 wt%). Both solutions were heated in the same manner as above. Typical stepwise radical polymerization of DNIG was also performed to obtain a DNIG formed by a two-step process (DNIG-2) shown in our previous paper [19].

### 2.2. Characterization of DNIG

Surface observation and elemental analysis of DNIG were performed by using a scanning electron microscope (SEM; JSM-7100F, JEOL, Tokyo, Japan) with energy-dispersive X-ray spectroscopy (EDX; JED-2300, JEOL). The thermal stability of DNIG was examined by thermogravimetric analysis (TG8120, Rigaku Co., Tokyo, Japan) under a nitrogen atmosphere to measure the weight loss from room temperature to 500 °C with a heating rate of 10 °C/min. We also examined the stability of DNIG against moisture. DNIG was placed in a Petri dish and then floated on the water bath in a closed environment. The water bath was heated at 50 °C for 48 h to provide moisture and water uptake was measured by thermogravimetric analysis. The mechanical strength of DNIG was examined through a compressive stress–strain test by using a universal testing system (Instron 3342, Instron). We set a cylindrical gel sample of 2 mm thickness and 4 mm diameter on the lower anvil to compress the upper anvil at 10%strain/min. Tribological properties of DNIG were examined by using a ball-on-plate type reciprocating tribometer (Tribogear type-38, Shinto Scientific Co. Ltd., Tokyo, Japan). A 10 mm *ϕ* ball sample was set in the upper holder connected to a load cell, and the sample gel substrate was fixed on a lower sliding stage. MEMP-TFSA was dropped onto the sample gel as a liquid lubricant and the friction force between a tribopair of ball and gel sample described as ball/sample was measured under 0.98 N at 0.5~50 mm/s to evaluate the lubrication properties. Glass, SUS304 and poly (tetrafluoroethylene) (PTFE) were chosen as counterface materials.

## 3. Results and Discussion

### 3.1. Physicochemical Properties of DNIG

#### 3.1.1. Structural and Chemical Analysis of DNIG

**Figure 1:**
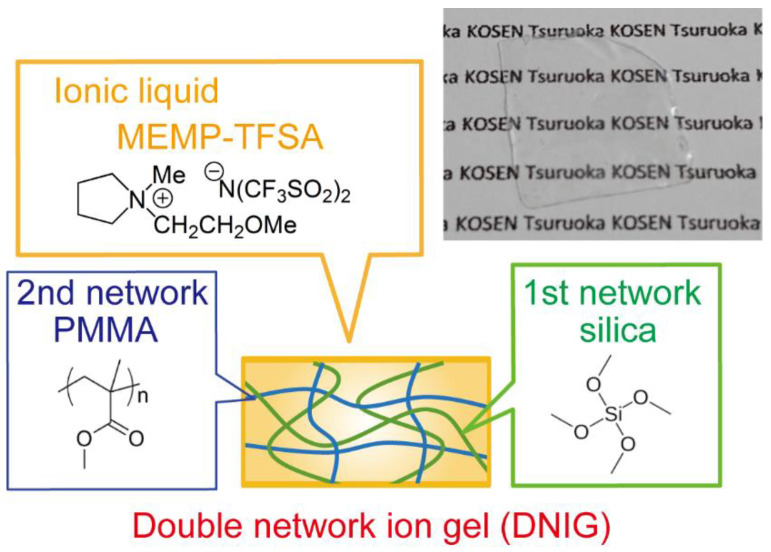
Schematic illustration of DNIG obtained by one-pot synthesis. A photograph of DNIG thin film (thickness: 200 µm) was also inserted in the upper right.

Figure 1 shows the chemical composition of DNIG-1 and its photograph. DNIG-1 could be easily fabricated as a thin film (thickness: 200 µm), which showed that the one-pot synthesis process can be applied as a simple coating process with a thin film of lubricant gel on the various surface. It should be noted that such a thin film cannot easily be obtained by the conventional stepwise radical polymerization process, where immediate swelling of the thin 1st gel film in the precursor solution of the 2nd network leads to the collapse of the gel itself.

**Figure 2:**
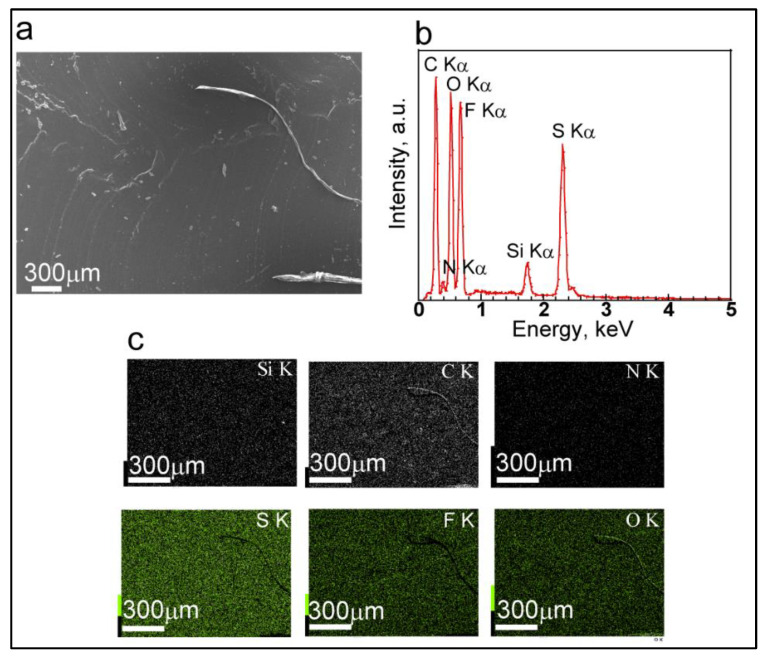
Surface SEM image (**a**), EDX spectra (**b**) and EDX mapping analysis data (**c**).

We next applied SEM and EDX to characterize the chemical composition of DNIG-1 (Figure 2). EDX mapping data show elements based on the 1st network silica (Si, O), 2nd network PMMA (C, O) and ionic liquid MEMP-TFSI (C, N, O, F, S). Although quantitative analysis of these components is difficult due to the overlap of elements, these mapping data support that each component was distributed evenly in DNIG.

#### 3.1.2. Thermal Stability of DNIG

**Figure 3:**
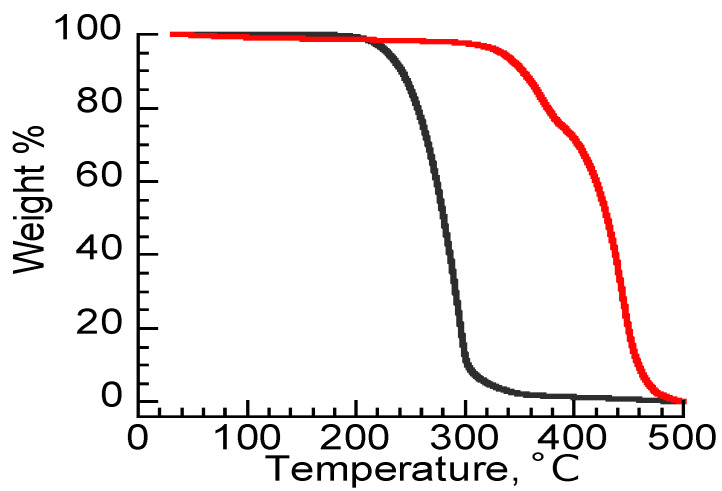
TGA curves of PAO (black line) and DNIG-1 (red line).

We examined the thermal stability of DNIG-1 from TGA curves and compared it with conventional lubricant, poly α olefine (PAO). Figure 3 shows the TGA curves of DNIG-1 and PAO. DNIG-1 did not show thermal degradation until 300 °C. The temperatures of 5% weight loss (*T*_5_) of DNIG-1 and PAO were 330 °C and 229 °C, respectively. Since DNIG-1 showed higher *T*_5_, DNIG-1 is expected to be an efficient gel lubricant at high temperatures. The obtained *T*_5_ of DNIG-1 was relatively higher than that of DNIG-2 (320 °C) [18], probably due to the higher thermal stability of the silica network compared with the 1st network of DNIG-2 (ionic liquid-type polymer). In addition, DNIG-1 also showed high resistance to moisture where the TGA curve did not show any change after exposure to 100% humidity for 48 h due to the hydrophobic property of MEMP-TFSA. These data show that DNIG-1 is expected to be utilized as a gel lubricant stable under high temperature or long time exposure where conventional oil lubricants cannot be applied.

#### 3.1.3. Mechanical Property of DNIG-1

**Figure 4:**
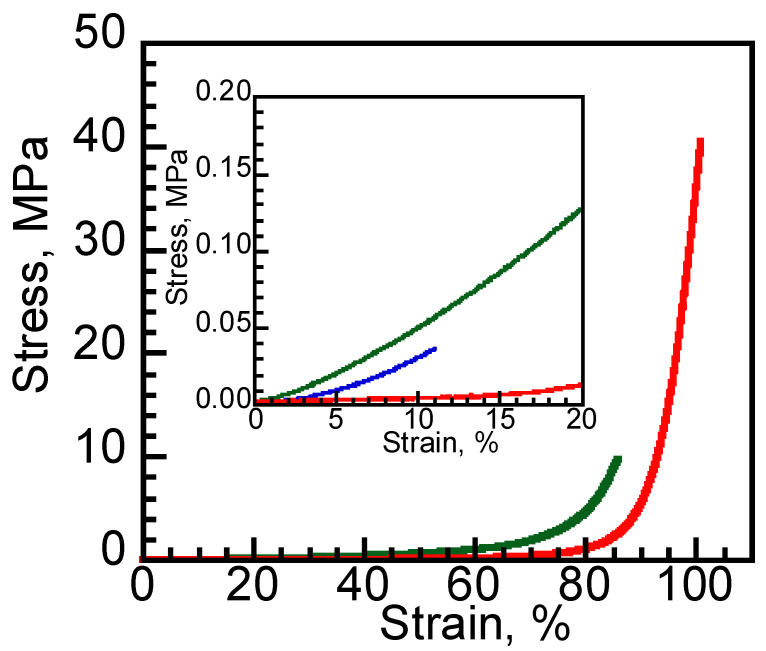
Compressive stress–strain curves of DNIG-1 (red line) and single network ion gel of silica (blue line) and PMMA (green line).

Compressive stress–strain curves were obtained to evaluate the mechanical properties of DNIG-1 (Figure 4). The fracture stress of DNIG-1(41 MPa) was much higher than that of single network gels obtained from TEOS (0.04 MPa) or MMA (10 MPa). It should be noted that the obtained value was higher than that of conventional DNIG-2 (30 MPa) obtained by the 2-step method. Kamio et al. showed that the mechanical strength of DNIG-1 is dominated by the microstructure of the 1st and 2nd networks [21]. When a silica nanoparticle network is synthesized before the 2nd polymer network, a spatially continuous silica nanoparticle network is formed to obtain DN structure. On the other hand, the fast formation of the 2nd polymer network works as a large diffusion barrier for the 1st network where silica nanoparticles form spatially dispersed clusters, resulting in µ-DN structure. They examined controlling polymerization rates of silica and PMMA networks by changing reaction temperature or radical initiator. As they examined the formation time of the 1st and 2nd networks by dynamic viscoelastic properties, the 1st network formed faster than the 2nd network when they applied a lower temperature with the AIBN initiator, which resulted in the formation of DN structure. Though it was difficult to compare directly the microstructure of DNIG with a different component, the obtained DNIG-1 in this study was supposed to possess DN structure since it showed higher mechanical strength than that of conventional DNIG-2.

#### 3.1.4. Lubrication Property of DNIG

**Figure 5:**
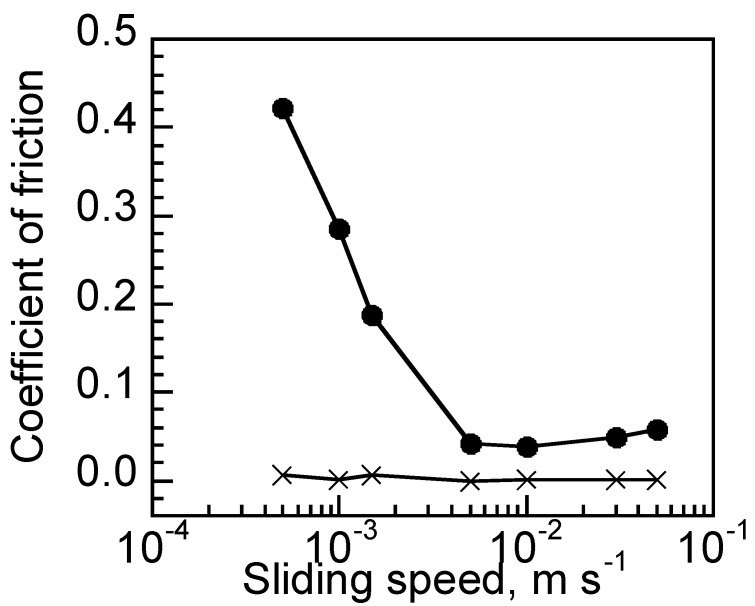
Sliding speed dependency of COF measured at glass/DNIG-1 (circle) and glass/DNIG-2 (triangle).

The lubrication properties of DNIG-1 and DNIG-2 were compared by sliding speed dependence of the coefficient of friction (COF). Both DNIG-1 and DNIG-2 show a decrease in COF as the sliding speed decreased under the load of 0.98 *N* (Figure 5). Such behaviors represent the lubrication regime of elastic or hydrodynamic lubrication regime in soft materials [13]. In the case of hydrodynamic lubrication, counterface materials are in contact so then viscous resistance is the dominant factor, where COF is constant under the same bearing characteristic number. However, COF from 50 to 10 mm/s of DNIG-1 was higher compared with that of DNIG-2 with the same lubricants, indicating that the lubrication regime of these gels is in the elastic lubrication regime.

The COF increased immediately when the sliding speed became lower than 5.0 mm/s, indicating that they are in the mixed lubrication regime where glass ball and DNIG were in partial contact. Lubrication behavior in the mixed lubrication regime is dominated by the ratio of viscous resistance and solid contact. DNIG-1 showed higher friction especially at slower sliding speeds, indicating that boundary lubrication between glass/DNIG-1 was much higher than that of glass/DNIG-2. As the 1st network derived from TEOS and glass ball are both composed of a SiO_2_ network, such high friction was supposed to be induced by high adsorption force due to the equal chemical potential of the same metal. We, therefore, changed the counterface material to examine the effect of the chemical composition of counterface materials on friction.

**Figure 6:**
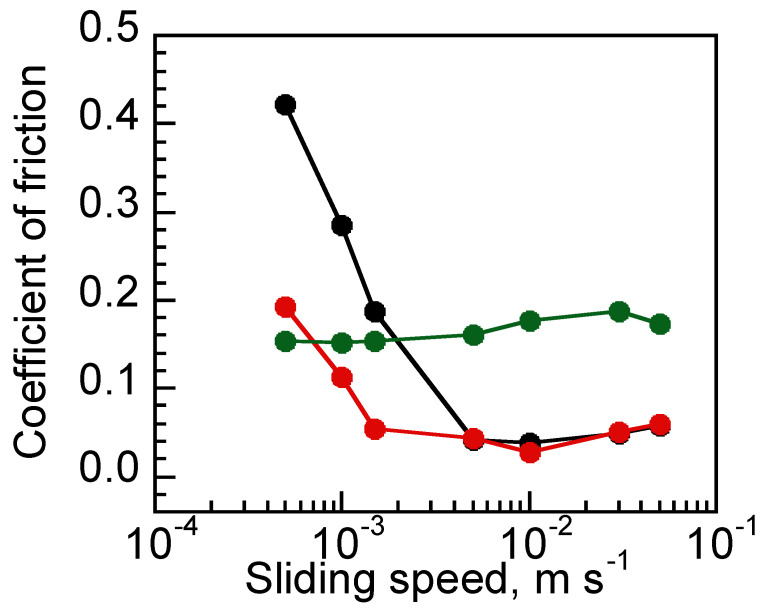
Sliding speed dependency of the COF measured at glass/DNIG-1 (black), SUS304/DNIG-1 (red) and PTFE/DNIG-1 (green).

Figure 6 shows the sliding speed dependency of COF at DNIG-1 and various counterface materials. While the COF of glass/DNIG-1 and SUS304/DNIG-1 showed similar values at a sliding speed of 50~5.0 mm/s, a drastic reduction in COF of SUS304/DNIG-1 was observed at a lower sliding speed (1.5~0.5 mm/s) compared with that of glass/DNIG-1. Such reduction of the COF supports the theory that the same metal of the tribopair resulted in high friction due to the higher adsorption force. In contrast, PTFE/DNIG-1 showed less dependent behavior against sliding speed, where COF was similar in the measured sliding speed range. Such behavior is typical in the case of the boundary lubrication regime, where liquid film between the contact surfaces is thin and solid contact was dominant in friction. As PTFE showed high contact angle to MEMP-TFSA compared with other counterface materials, such behavior was supposed to be based on repellent PTFE surface against MEMP-TFSA. Such a repellent surface decreases the thickness of the liquid film of lubricant to induce the shift from a mixed to boundary lubrication regime, which resulted in high friction at a wide sliding speed range due to the increase of solid contact. Additionally, PTFE/DNIG-1 showed lower friction at 0.5 mm/s compared to glass/DNIG-1, indicating that boundary lubrication was lower in the case of PTFE/DNIG-1. The obtained result was supposed to be due to the self-lubrication effect of PTFE where worn particles of PTFE attached to the DNIG-1 surface to form the PTFE layer, inducing low friction of PTFE/PTFE [22].

## 4. Conclusions

One-pot synthesis of double network ion gel was successfully achieved by combining polycondensation of TEOS and radical polymerization of MMA. SEM and EDX mapping data of the obtained DNIG-1 supported that each component was distributed evenly in DNIG-1. TGA data show that DNIG-1 is expected to be utilized as a gel lubricant that is stable under high temperature or long time exposure where conventional oil lubricants cannot be applied. Compressive stress–strain curves showed that DNIG-1 obtained by one-pot synthesis possesses high compressive fracture stress (41 MPa) compared with that obtained by conventional two-step synthesis (30 MPa). The COF of the glass/DNIG-1 tribopair was relatively higher than that of glass/DNIG-2, probably due to the melting effect between the same metals. Change of counterface materials effectively reduced the COF at DNIG-1 which suggests the importance of the appropriate selection of tribopair. The obtained results open up the way for facile fabrication of lubricant gel with high mechanical strength and thermal stability for industrial application such as lubricant gel seal possessing both low friction and good sealing property.

## Data Availability

Data is contained within the article.
